# Endocrine Profiling and Prioritization of Environmental Chemicals Using ToxCast Data

**DOI:** 10.1289/ehp.1002180

**Published:** 2010-09-08

**Authors:** David M. Reif, Matthew T. Martin, Shirlee W. Tan, Keith A. Houck, Richard S. Judson, Ann M. Richard, Thomas B. Knudsen, David J. Dix, Robert J. Kavlock

**Affiliations:** 1 National Center for Computational Toxicology, Office of Research and Development, U.S. Environmental Protection Agency, Research Triangle Park, North Carolina, USA;; 2 Office of Science Coordination and Policy, Office of Pollution Prevention, Pesticides and Toxic Substances, U.S. Environmental Protection Agency, Washington, DC, USA

**Keywords:** androgen, chemical prioritization, data integration, endocrine disruption, estrogen, screening, ToxCast, toxicity profile, ToxPi

## Abstract

**Background:**

The prioritization of chemicals for toxicity testing is a primary goal of the U.S. Environmental Protection Agency (EPA) ToxCast™ program. Phase I of ToxCast used a battery of 467 *in vitro*, high-throughput screening assays to assess 309 environmental chemicals. One important mode of action leading to toxicity is endocrine disruption, and the U.S. EPA’s Endocrine Disruptor Screening Program (EDSP) has been charged with screening pesticide chemicals and environmental contaminants for their potential to affect the endocrine systems of humans and wildlife.

**Objective:**

The goal of this study was to develop a flexible method to facilitate the rational prioritization of chemicals for further evaluation and demonstrate its application as a candidate decision-support tool for EDSP.

**Methods:**

Focusing on estrogen, androgen, and thyroid pathways, we defined putative endocrine profiles and derived a relative rank or score for the entire ToxCast library of 309 unique chemicals. Effects on other nuclear receptors and xenobiotic metabolizing enzymes were also considered, as were pertinent chemical descriptors and pathways relevant to endocrine-mediated signaling.

**Results:**

Combining multiple data sources into an overall, weight-of-evidence Toxicological Priority Index (ToxPi) score for prioritizing further chemical testing resulted in more robust conclusions than any single data source taken alone.

**Conclusions:**

Incorporating data from *in vitro* assays, chemical descriptors, and biological pathways in this prioritization schema provided a flexible, comprehensive visualization and ranking of each chemical’s potential endocrine activity. Importantly, ToxPi profiles provide a transparent visualization of the relative contribution of all information sources to an overall priority ranking. The method developed here is readily adaptable to diverse chemical prioritization tasks.

In 1996, the U.S. Congress passed two laws affecting the regulation of pesticides and other chemicals. Both of these laws, the Food Quality Protection Act of 1996 (FQPA 1996) and the Safe Drinking Water Act Amendments of 1996 (SDWA Amendments 1996), contained provisions for assessing the potential for chemicals to interact with the endocrine system. The FQPA (1996) required the U.S. Environmental Protection Agency (EPA) to

develop a screening program, using appropriate validated test systems and other scientifically relevant information, to determine whether certain substances may have an effect in humans that is similar to an effect produced by a naturally occurring estrogen, or such other endocrine effect as the Administrator may designate.

Chemicals identified for testing under the two statutes include all pesticide chemicals (both active and inert ingredients in pesticide formulations), as well as any other substances that may have an effect that is cumulative with effects of a pesticide if the administrator determines that a substantial population is exposed to such a substance. Furthermore, the SDWA Amendments (1996) state that

In addition to the substances referred to in . . . the Federal Food, Drug, and Cosmetic Act (21 U.S.C. 346a(p)(3)(B)) . . ., the Administrator may provide for testing under the screening program authorized by [the FFDCA] . . . of any other substance that may be found in sources of drinking water if the Administrator determines that a substantial population may be exposed to such substance.

Based largely on recommendations from the Endocrine Disruptor Screening and Testing Advisory Committee (EDSTAC), a U.S. EPA advisory committee convened to recommend approaches to addressing the requirements of the FQPA (Gray 1998), the U.S. EPA’s Endocrine Disruptor Screening Program (EDSP) developed and validated assays to be used in a two-tiered screening approach whose initial focus is on the estrogen, androgen, and thyroid (E, A, and T) systems in mammals, along with select ecological species. The Tier 1 battery assesses the potential of a chemical to interact with the endocrine system (specifically E, A, and T), and the Tier 2 assays are intended to identify possible effects within these systems. The Tier 1 battery consists of 11 assays: an estrogen receptor (ER) binding assay, an ER transactivation assay, an androgen receptor (AR) binding assay, an aromatase assay, a steroidogenesis assay, a rat uterotrophic assay, a rat Hershberger assay, rat pubertal male and female assays, a frog metamorphosis assay, and a fish partial life cycle assay (U.S. EPA 2009). The U.S. EPA has estimated the cost of conducting and reporting the tests in this first tier to be approximately half a million U.S. dollars (U.S. EPA 2007). Because of the lack of reliable high-throughput screening (HTS) methodologies available at the time, a decision was made to prioritize the initial list of 67 chemicals to be screened using the Tier 1 battery based solely on estimates of potential for human and environmental exposures (U.S. EPA 2009). However, the same document also states that the U.S. “EPA anticipates that it may modify its chemical selection approach for subsequent screening lists” based upon considerations including “the availability of new priority-setting tools (e.g., High Throughput Prescreening or Quantitative Structure Activity Relationships models).” Issuance of test orders for this Tier 1 screening began in 2009 (U.S. EPA 2010a).

ToxCast is a large-scale experiment using a battery of *in vitro* HTS assays to develop activity signatures predicting the potential toxicity of environmental chemicals at a cost that is < 1% of that required for full-scale animal testing (Dix et al. 2007). Phase I included 467 assays across nine technology platforms (Judson et al. 2010). Assays include both cell-free (biochemical) and cell-based measures, largely using human cells or cell lines, and cover a wide spectrum of biological targets or effects, including cytotoxicity, cell growth, genotoxicity, enzymatic activity, receptor binding, ion channels, transcription factor activity and downstream consequences, gene induction, and high-content imaging of cells. A major goal of ToxCast is to provide U.S. EPA regulatory programs with information helpful in prioritizing chemicals for more detailed toxicological evaluations. These detailed evaluations may include nontraditional, mechanism-focused *in vivo* or *in vitro* tests. The rational prioritization of chemicals is anticipated to enable more efficient use of animal resources in toxicity testing.

Of particular relevance to the EDSP, roughly 15% of the ToxCast assays examine various aspects of the estrogen (six assays), androgen (five assays), and thyroid (five assays) signaling pathways, as well as a number of other nuclear receptor (NR) pathways (e.g., glucocorticoid receptor, peroxisome proliferator–activated receptor, pregnane X receptor; 36 assays) and xenobiotic-metabolizing enzymes (XMEs; cytochrome P450s, including aromatase; 38 assays) that have potential relevance to endocrine signaling. Because phase I of ToxCast capitalized on the richness of traditional toxicology information available for food-use pesticides (Knudsen et al. 2009; Martin et al. 2009a, 2009b), a large fraction (52 of 67) of the list of chemicals for initial EDSP Tier 1 testing were included in the HTS screening.

In this article, we describe the endocrine profiles for the entire ToxCast library of 309 unique chemicals, with particular focus on those chemicals designated for the first round of EDSP screening. Importantly, we provide a flexible ranking system by which chemicals may be prioritized for screening in the more expensive animal-based studies or other, lower-throughput testing methods. We define the Toxicological Priority Index (ToxPi^TM^) as a tool for objective chemical prioritization based upon formal integration across multiple domains of information. The ToxPi framework provides a graphical system for analyzing complex toxicological data that was designed with several key features in mind: extensibility to incorporate additional types of data (e.g., measures of biotransformation, exposure, dosimetry); multivariate assessment of toxicity relative to any set of chemicals; differential weighting factors for various information domains and data sources; transparency in score derivation and visualization; and flexibility to customize components for diverse prioritization tasks. We assessed the validity of our prioritization system by exploring the distribution of well-characterized chemicals within the ToxPi rankings, inspecting profiles within structurally homogeneous chemical classes, and assessing sensitivity to spurious assay results. The results demonstrate that integrating data across partially redundant assays and multiple knowledge domains gives a robust priority rank across chemicals and suggest how this information can be combined in a transparent manner to prioritize further testing.

## Materials and Methods

### Data sources

The data used to develop the prioritization profiles for the 309 unique chemicals are housed in U.S. EPA’s ToxMiner database, an internal repository for assay data from ToxCast, and have been previously described in detail (Judson et al. 2010). Briefly, data were gathered from 467 assays using a variety of technologies, including biochemical HTS (Judson et al. 2010) and cell-based HTS assays measuring direct molecular interactions with specific protein targets (Inglese et al. 2007); high-content cell imaging assays measuring complex cellular phenotypes (Giuliano et al. 2006); a multiplexed gene expression assay for XMEs and transporters in human primary hepatocytes (LeCluyse et al. 2005); and multiplexed transcription factor reporter assays (Martin et al. 2010; Romanov et al. 2008). For the present study, we used specific experience with the ToxCast assays in combination with expert knowledge to compartmentalize the data in a manner informative to assessing endocrine-related activity. This resulted in a subset of 90 assays having putative endocrine relevance, divided into five broad categories: AR, ER, thyroid receptor (TR), XME/ADME (absorption, distribution, metabolism, and excretion), and other NRs [for a categorized listing of and additional details on all assays, see Supplemental Material, Tables 1 and 2, respectively (doi:10.1289/ehp.1002180)]. Some of these assays were considered to be directly related to the types of measures being collected in Tier 1 of the EDSP. Other assays against a number of NRs (38 other NR assays) and human and rat cytochrome 450s (36 XME/ADME assays) were included as potentially reflecting either direct (e.g., inhibition of aromatase activity) or indirect (e.g., alterations in metabolism affecting synthesis or degradation of endogenous hormones) effects on the endocrine system *in vivo*.

For all *in vitro* assays, we calculated a characteristic effective concentration (micromolar) for each chemical–assay combination as described by Judson et al. (2010). These values were half-maximal activity concentrations (AC_50_) for all assays except the multiplexed transcription factor assay, for which lowest effective concentrations (LECs) were calculated. Chemical–assay combinations that did not show activity below the highest concentration tested were labeled inactive. The complete data set, including AC_50_/LEC values for all chemical–assay measurement pairs, is available from the U.S. EPA ToxCast web site (U.S. EPA 2010b).

The two chemical properties we used were a derived octanol/water partition coefficient (log*P*) and a predicted Caco-2 (cell membrane permeability assay) descriptor, which were intended to provide measures of bioavailability (related to gastrointestinal absorption and permeability, respectively, as information that would not have been captured by the *in vitro* assays). The log*P* descriptor was calculated using LeadScope (Leadscope Inc., Columbus, OH). The predicted percent human absorption or Caco-2 descriptor was calculated using QikProp software (version 3.3.021; Schrödinger, New York, NY). In cases where QikProp was unable to calculate a value, an interpolated *P*_Caco_ prediction was used:


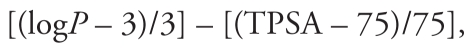


where TPSA is total polar surface area calculated using LeadScope.

The pathway information capitalized on data in external knowledge bases. As described by Judson et al. (2010), *in vitro* targets were mapped to human genes as an intermediate connection between assays and pathways. From these assay–gene–pathway connections, chemicals showing activity in at least five assays that mapped to a given pathway were assigned a “pathway perturbation score” as the minimum AC_50_/LEC value among the assays mapped to that pathway. The pathways used were selected for endocrine relevance from the Kyoto Encyclopedia of Genes and Genomes (KEGG) (Ogata et al. 1999), Ingenuity software (version 8.7; Ingenuity Systems Inc., Redwood City, CA), and the Online Mendelian Inheritance in Man repository (National Center for Biotechnology Information 2010). The list of 27 specific pathway components used is given in Supplemental Material, Table 1 (doi:10.1289/ehp.1002180), and details of each component (as stored in the ToxMiner database) are given in Supplemental Material, Table 2. The pathway slices thus represent knowledge-based aggregations of individual assay results and serve to highlight bioactivity in cases where singular assay components demonstrated only low-to-moderate potency.

### Rationale, notation, and definition of ToxPi

**Figure 1:**
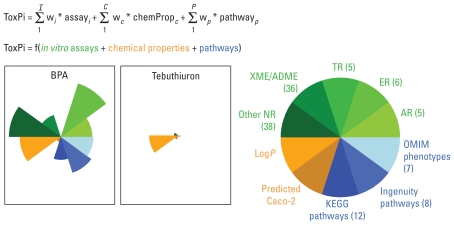
Definitions and notation for ToxPi. Weighted combinations of data were integrated for each chemical from multiple domains, with relative scores represented in ToxPi profiles composed of slices based on one or more components. Domains are basic data types represented by slices of a given color family: green, *in vitro* assay slices; orange, chemical properties; blue, pathways. Slices represent data from related assays, properties, or pathways, including AR, ER, TR, and seven other slices (see “Materials and Methods” for a full description). Ninety assays, two properties, and 27 pathways make up the 119 components of this endocrine ToxPi (e.g., the ERα transcription factor assay is one of six components in the ER slice). The number of components in each slice is shown in parentheses. ToxPi profiles for bisphenol A and tebuthiuron are shown as examples of high- and low-ranked chemicals.

The framework for our profiling and prioritization system is detailed in Figure 1. For each chemical, ToxPi, a dimensionless index score, is calculated as a weighted combination of all data sources that represents a formalized, rational integration of information from different domains. Visually, ToxPi is represented as component slices of a unit circle, with each slice representing one piece (or related pieces) of information (Figure 1). For each slice, distance from the origin (center) is proportional to the normalized value (e.g., assay potency or predicted bioavailability) of the component data points composing that slice, and the width (in radians) indicates the relative weight of that slice in the overall ToxPi calculation. For example, in Figure 1, the slice representing ER assays for bisphenol A (BPA) extends farther from the origin than the corresponding slice for tebuthiuron, indicating that BPA is more potent across ER assays than is tebuthiuron. In the implementation presented here, all 10 slices are weighted equally in the overall ToxPi calculation, so the graphical width of all slices is equal to the angle, θ, formed by dividing 2π radians into 10 sections, or 2π/10 = π/5 radians = 36 degrees.

**Figure 2:**
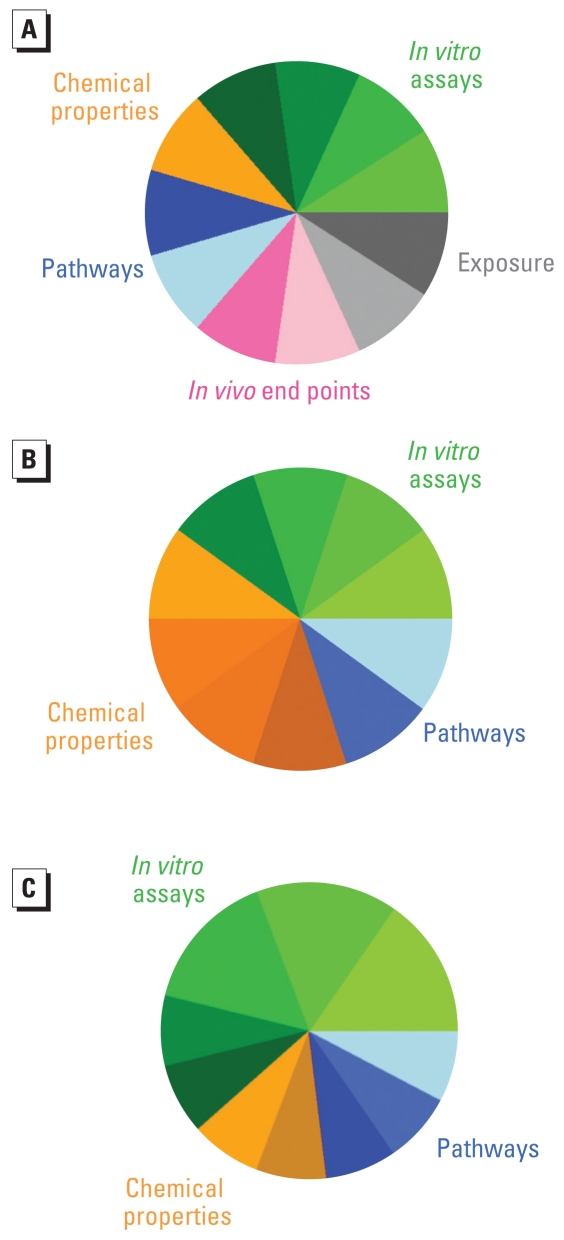
Alternative ToxPi implementations. Prioritization tasks might (*A*) incorporate additional components and slices from other domains (e.g., consideration of exposure potential); (*B*) customize individual domains (e.g., add a targeted set of chemical descriptors); or (*C*) adjust weighting schemes according to specific prioritization tasks, or based on component, slice, or domain meaning [e.g., weights (w*_i_* = 1, 2, 3) of *in vitro* assay slices 1, 2, and 3 (representing AR, ER, and TR, respectively) have been increased].

Alternative weighting schemes that differentially emphasize individual ToxPi slices would therefore be graphically represented by different angular widths corresponding to the weight of each slice. Figure 2 details some alternative implementations for hypothetical prioritization tasks within the general ToxPi framework. In Figure 2A, additional domains have been included to represent knowledge from *in vivo* study results [e.g., from the Toxicity Reference Database (ToxRefDB) multigenerational studies (Martin et al. 2009b)] and exposure estimates [e.g., from the Simple Exposure Tool (SimET) database (Health Canada 2008)]. This could represent a scheme for prioritization of chemicals with respect to particular health outcomes, such as liver carcinogenicity. In Figure 2B, additional chemical descriptors have been included. This could represent a collection of quantitative structure–activity relationship (QSAR) models or a specialized prioritization task such as developmental toxicity, wherein a targeted set of chemical properties related to placental transport would be important. In Figure 2C, the components shown are the same as in Figure 1, but the weights of the AR, ER, and TR slices have been increased. This reweighting could reflect a hypothesis that the *in vitro* assays measuring the AR/ER/TR axis should have greater influence for prioritizing endocrine disruptors.

### Implementation of a profiling and prioritization methodology for endocrine disruptors

Here, we implement a ToxPi formulation that is specific to the task of endocrine prioritization by selecting data sources having putative endocrine relevance (see “Data sources,” above). The function developed for creating ToxPi profiles was based upon R code (R Development Core Team 2008) in the *graphics*, *gdata*, and *lattice* packages (R Foundation for Statistical Computing 2010), and the visualization is a modification of iconographic displays called “spider” or “radar” plots (von Mayr 1877). The slices are defined in Figure 1, and the components of each slice are given in Supplemental Material, Table 1 (doi:10.1289/ehp.1002180). Because of the screening aspect of this prioritization task, we have been inclusive with the set of assays and pathways chosen to ensure that we capture as many aspects of endocrinology as possible within the confines of the data. This inclusiveness in the screening is designed to minimize false negatives.

**Figure 3:**
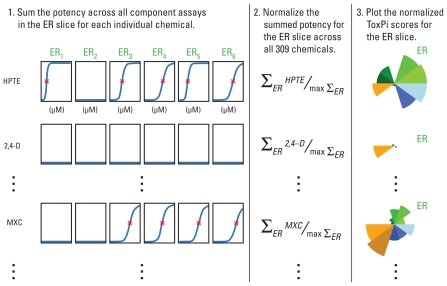
Translation of results into ToxPi score profiles. The concentration–response curves for each of the six assays in the ER slice are shown for three example chemicals. On each curve, the red asterisk represents the AC_50_ (potency) for assay “hits,” and flat blue lines indicate assays that are inactive for that chemical. For nonassay slices, the same procedure is followed, with AC_50_ values replaced by particular chemical property values, pathway scores, and so forth. Abbreviations: 2,4-D, 2,4-dichlorophenoxyacetic acid; max, maximum; MXC, methoxychlor.

Figure 3 is a schematic that details how data are translated into ToxPi scores; Supplemental Material, Table 3 (doi:10.1289/ehp.1002180) provides slice-wise scores for all chemicals. For this application, the chemical-wise slice results are normalized to the interval [0,1] by dividing each chemical result by the slice maximum, where results represent relative potency (*in vitro* assays), bioavailability (chemical properties), or perturbation score (pathways). Values closer to the unit score (equal to 1) translate to higher potency, higher predicted bioavailability, or greater pathway perturbation relative to all other chemicals. Conversely, values closer to the origin (equal to 0) translate to lower potency, lower bioavailability, and lesser pathway perturbation across the corresponding domains. Values at zero (i.e., slices not extending at all from the origin) translate to “inactive/no activity.” As conveyed by the equal radial width of all slices in Figure 1, the slices are not differentially weighted for this implementation. However, by using a smaller number of targeted components in the AR, ER, and TR slices, individual component assays within these slices exert a greater influence over that slice’s composite score than do individual assays from one of the larger slices for other NRs or XME/ADME.

## Results

### ToxPi profiles of the initial EDSP Tier 1 chemicals

For a broad view of the EDSP chemicals in the context of the entire ToxCast phase I set, Supplemental Material, Figure 1 (doi:10.1289/ehp.1002180) shows the ToxPi profiles for all chemicals, with the initial EDSP Tier 1 chemicals highlighted by red frames. The total colored area for each chemical signifies its overall ToxPi score. Closer inspection of individual chemical profiles reveals the reason(s) underlying overall scores. For example, the known AR modulators vinclozolin (Martinovic et al. 2008) and linuron (Cook et al. 1993) have prominent AR slices (see Supplemental Material, Figure 1). Linuron, which has been shown to alter thyroid hormone concentrations *in vivo* (O’Connor et al. 2002), also showed some activity within the ToxPi TR slice. Inspection of the underlying ToxCast assay data reveals that linuron stimulated expression of UGT1A1 (UDP glycosyltransferase 1 family, polypeptide A1) in human hepatocytes, which is consistent with emerging evidence that thyroid hormone concentrations are related to signaling events in the liver (Ding et al. 2006; O’Connor et al. 2002). This result illustrates the value of capturing information from a broad range of *in vitro* assays, probing a variety of mechanistic pathways, when screening chemicals with heterogeneous modes of action.

In the dot plot shown in Supplemental Material, Figure 2 (doi:10.1289/ehp.1002180), the chemicals are sorted according to ToxPi, from highest score to the lowest, showing that the initial EDSP Tier 1 chemicals are distributed throughout the ToxPi-sorted distribution. This is not surprising because these were selected based solely upon exposure considerations. The obvious advantage of the multidomain ToxPi is that expanding information coverage can fill holes in the screening net, thus increasing the likelihood of detecting true endocrine-active chemicals in the EDSP battery and augmenting efficiency by ensuring optimal use of screening and testing resources.

### Exploring the distribution of reference chemicals within the ToxCast set

**Figure 4:**
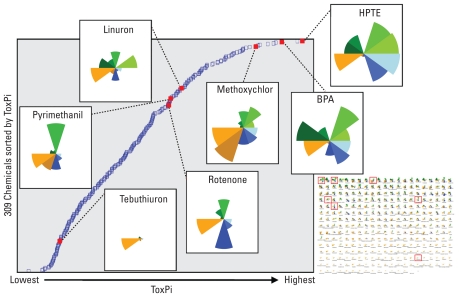
Distributional dot plot showing reference chemicals along the distribution of sorted ToxPi scores for all 309 ToxCast phase I chemicals. Each blue square indicates the ToxPi for one chemical, sorted according to overall ToxPi; the ToxPi for each reference chemical is indicated by a solid red square. From highest to lowest ToxPi, the reference chemicals are HPTE, BPA, methoxychlor, linuron, pyrimethanil, rotenone, and tebuthiuron. The inset shows the sorted ToxPi profiles for all chemicals (starting with the highest ToxPi in the upper left and proceeding to the lowest in the bottom right), with reference chemicals highlighted by solid red boxes. These profiles have been translated into the distributional dot plot described above.

Until EDSP Tier 1 assay results are available, the best evaluation of our ToxPi implementation for endocrine prioritization is to assess the relative distribution of “reference” chemicals in the context of specific slices. Reference chemicals are those for which we have a substantial body of evidence in support of hypotheses regarding their toxicological activities. Figure 4 shows the individual ToxPi profiles for these reference chemicals and their positions along the sorted ToxPi distribution for all 309 chemicals. For assessing ToxPi, the list of reference chemicals should be spread throughout the score distribution to make sure that our ranking does not relegate known hazards to the bottom of the list. Methoxychlor and its metabolite 2,2-bis-(*p*-hydroxyphenyl)-1,1,1-trichloroethane (HPTE) are among the chemicals with the highest ToxPi. These chemicals both have estrogenic effects (Miller et al. 2006), and in ToxCast data, the daughter metabolite (HPTE) demonstrated higher potency across more assays than did its parent, consistent with evidence showing that the metabolism of methoxychlor to HPTE results in higher ER affinity (Nimrod and Benson 1997). BPA is known to bind ER (Matthews et al. 2001) and is a known ER agonist (Chapin et al. 2008) that was also active across multiple ToxCast ER assays. However, BPA’s relatively high overall ToxPi score is also due to its activity relative to AR, other NRs, XME/ADME (including the aromatase biochemical HTS assay), and the endocrine-relevant pathway domains. In the literature, BPA has shown both estrogenic properties and the ability to act as an antiandrogen and an aromatase inhibitor (Bonefeld-Jørgensen et al. 2007). Linuron, which is an antiandrogen and has been associated with androgen-related reproductive effects in rats, has a prominent AR slice in our data due to activity in the cell-based HTS assay for AR antagonism plus the biochemical AR binding assay (Lambright et al. 2000). Pyrimethanil, which has been shown to stimulate thyroid hormone metabolism (Hurley 1998), and rotenone, which is known to modulate thyroid hormone levels, demonstrated activity within the TR slice [via the transcription factor reporter assay for *THRa1* (thyroid hormone receptor-α) and *UGT1A1* gene expression] and activity across multiple KEGG pathways, including thyroid cancer (Wang et al. 2004). Tebuthiuron showed almost no activity across endocrine-related assays and lacked effects in multigenerational studies recorded in the ToxRefDB (Martin et al. 2009b), indicating that the relatively low ToxPi for this chemical is appropriate.

### Assessing ToxPi stability

**Figure 5:**
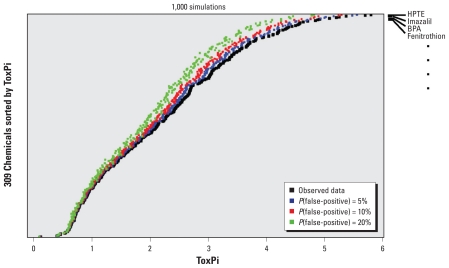
Assessment of the stability of ToxPi rankings in the presence of spurious assay results. The simulated probability of a spurious result on a given component assay ranges from 5% to 20%. Results for chemProp (orange) slices were held constant because they do not necessarily represent stochastic assays. Each data point shows the mean simulated ToxPi under each condition. For all simulation conditions, the chemicals are ordered according to the overall ToxPi score in the observed data (i.e., HPTE is line 1, BPA is line 2, and so forth for all 309 lines).

For any set of assays, false-positive results are a concern. Therefore, to assess the robustness of the ToxPi rankings (prioritization) presented here, we performed a simulation study testing sensitivity to spurious assay results. Simulations were designed to test the sensitivity of the ToxPi ranking for varying levels of false-positive results across all slices. For each simulation, we applied the given binomial false-positive rate to the observed component assay results. Figure 5 presents the mean ToxPi score of each chemical across 1,000 simulations each for 5%, 10%, and 20% false-positive probability. These results show that, even in the face of relatively high (20%) error rates, the overall ToxPi score is relatively stable. We observed the same result in concurrent false-negative simulations (data not shown). More important, the relative priority ranking given by ToxPi (i.e., resorting the chemicals according to one of the simulated distributions) is reasonably robust: In situations where a chemical’s absolute rank changes, it tends to swap positions with a neighbor. This is in contrast to the large shifts in relative rank that would occur in a prioritization scheme reliant on singular pieces of information, wherein individual errors would markedly shift the relative ranks. These simulation results demonstrate a major strength of integrating multiple pieces of information to achieve stable prioritizations. Also, because ToxPi is intended to be used as an index for ranking (as opposed to an absolute threshold), adding or removing data from particular assays will not dramatically alter prioritization—given sufficient collections of component information. For most conceivable uses, absolute rankings will be less important than quantile regions along the entire ToxPi distribution (e.g., chemical 4 and chemical 8 will still be in the top 5%).

### Inspecting profiles within structurally homogeneous chemical classes

Inspecting profiles within related chemical classes is informative for assessing the utility of ToxPi applications. Supplemental Material, Figure 3 (doi:10.1289/ehp.1002180) includes profiles for all 12 triazole fungicides present in ToxCast phase I. From the ToxPi profiles, this group of chemicals has similar log*P* values, similar Ingenuity pathway perturbation scores, and similar scores on the XME/ADME slice. However, the chemicals vary in the AR, ER, and TR slices, which agrees with evidence that individual triazoles differ widely with respect to reproductive toxicity observed *in vivo* (Goetz et al. 2007).

## Discussion

ToxPi is an innovative and integrative approach to incorporating multidimensional information into a flexible and transparent system for the prioritization of chemicals for future toxicological testing. Concurrent with the quantitative integration across domains of information, the profiles visually summarize the underlying prioritization rationale by explicitly showing how each piece of information contributes to the overall score. This initial implementation of the ToxPi framework indicates that an integrated approach, wherein multiple domains of toxicological knowledge are simultaneously incorporated into chemical prioritization, can reasonably rank the ToxCast phase I chemicals for observed and potential endocrine-related toxicity. These rankings represent a systematic rationale for informing chemical prioritization decisions. Tangential to the main prioritization goal, ToxPi profiling in combination with task-specific reference chemicals may also be useful for highlighting data needs relative to existing risk assessment information.

A salient advantage of the ToxPi framework is its flexibility to incorporate information from new domains and to be continuously updated with new chemical data. For example, beyond the 119 components we have included in this implementation, we would like to add additional components, such as QSAR predictions for ER-binding potential (Schmieder et al. 2003). Ideally, future implementations could include an exposure domain. As stated above, the prioritization ranks presented here will differ from the EDSP Tier 1 prioritized list because we do not have consistent exposure data for the entire chemical set. Despite the fact that simulations show ToxPi to be robust to component errors and resistant to spurious shifts in relative rank, inclusion of slices wherein a preponderance of chemicals have missing data could bias prioritizations toward those having that data.

The *in vivo* studies included in the current version of ToxRefDB may not be especially sensitive for some endocrine effects because most of the multigeneration reproductive studies were conducted using older test guidelines with limited coverage of endocrine-sensitive phenotypes (Stoker and Kavlock 2010). In addition, a number of chemicals highly ranked by ToxPi either have no study data in ToxRefDB or have only weak associations with endocrine-related end points. This indicates that ToxCast applications such as ToxPi data will not only support the prioritization of chemicals yet to be tested for reproductive or endocrine effects but also help identify previously tested chemicals with unrecognized data gaps.

Besides data concerns, a number of statistical avenues are yet to be explored. In cases where a definitive priority order of chemicals is known *a priori*, weight optimization could be carried out using a Bayesian method. Such optimization may also be possible for a definitive reference chemical set, such as when Tier 1 EDSP data become available. In the future, particular ToxPi slices could also be used to represent complex components, such as predictive signatures developed as part of ToxCast or other multivariate models. Another use of the ToxPi approach is to evaluate the ToxCast assays as applied to endocrine prioritization or screening. Known data gaps in the current assay suite include biological targets in the hypothalamic–pituitary–gonadal axis, which are covered in the EDSP screening battery. Further ToxCast efforts will attempt to fill these data gaps for mechanisms or toxicity pathways that are not captured at present. Because it is currently impossible for any screening system to cover every conceivable toxicity mechanism in the face of species differences, biotransformation, the nonexistence of “perfect” assays, and other complicating issues, there will always be gaps in our screening capabilities. However, key pathways of endocrine disruption are relatively well defined, and a number of HTS assays are available to cover many of those pathways. Given the need to prioritize large numbers of chemicals for expensive animal-based bioassays, applying ToxCast results toward the identification of chemicals with high likelihoods of interacting with these pathways is a logical prioritization step. We hope that an integrated framework such as ToxPi will help bring together data from alternative, mutually complementary sources to inform and guide rational prioritization decisions.

Last, it is important to recognize the fluid nature of prioritization tasks. The optimal prioritization would take into account both *a*) measured or computed data that reflect inherent properties of chemicals in relation to biological systems, and *b*) regulatory considerations that factor in human-activity–based chemical-use patterns and exposure metrics. Whereas the former type of data can be objectively measured and anchored to biological systems and outcomes, the latter are dependent on production patterns, environmental fate and transport, product use, and human activities. Hence, a prioritization approach such as ToxPi, when implemented in the broadest sense, incorporates both objective chemical and biological data that are amenable to external validation, and other considerations such as use and exposure that can be validated only in terms of a predefined subjective framework. These fundamentally different factors must be recognized and taken into account in any effort to validate implementations of the current prioritization approach. For example, a metric of validation for the present implementation, which relies primarily on *in vitro* assay data and chemical properties for predicting toxicity, would be in the context of actual toxicity measures. However, because the initial EDSP Tier 1 chemicals were chosen in large measure because of exposure considerations, a prioritized ranking based on toxicity measures alone would not be expected to coincide precisely.

## Conclusions

The ToxPi profiles developed here provide graphical insight into the relative contributions of multiple data domains considered in this chemical profiling and prioritization. It is amenable to incorporating extant prioritization schemes and relevant data from diverse sources, thereby facilitating meta-analysis across resources from the U.S. EPA and elsewhere. Because ToxPi scores are intended for relative ranking, particular implementations of this framework can be continually updated with new chemicals and future data. A framework amenable to data growth will be vital as the body of chemical information grows exponentially with efforts such as REACH (Registration, Evaluation, Authorisation and Restriction of Chemical substances) in the European Union (EC 1907/2006), subsequent phases of the U.S. EPA’s ToxCast program, and the inclusion of information as it becomes available from the EDSP test batteries.
